# News exposure predicts anti-Muslim prejudice

**DOI:** 10.1371/journal.pone.0174606

**Published:** 2017-03-31

**Authors:** John H. Shaver, Chris G. Sibley, Danny Osborne, Joseph Bulbulia

**Affiliations:** 1 Religion Programme, University of Otago, Dunedin, New Zealand; 2 School of Psychology, University of Auckland, Auckland, New Zealand; 3 Faculty of Humanities and Social Sciences, Victoria University of Wellington, Wellington, New Zealand; Universiteit van Amsterdam, NETHERLANDS

## Abstract

News coverage of Islamic extremism is reigniting debates about the media’s role in promoting prejudice toward Muslims. Psychological theories of media-induced prejudice date to the 1950’s, and find support from controlled experiments. However, national-scale studies of media effects on Muslim prejudice are lacking. Orthogonal research investigating media-induced prejudice toward immigrants has failed to establish any link. Moreover, it has been found that people interpret the news in ways that confirm pre-existing attitudes, suggesting that media induced Muslim prejudice in liberal democracies is unlikely. Here, we test the association between news exposure and anti-Muslim prejudice in a diverse national sample from one of the world’s most tolerant societies, where media effects are least likely to hold (*N* = 16,584, New Zealand). In support of media-induced Islamophobia, results show that greater news exposure is associated with both increased anger and reduced warmth toward Muslims. Additionally, the relationship between media exposure and anti-Muslim prejudice does not reliably vary with political ideology, supporting claims that it is widespread representations of Muslims in the news, rather than partisan media biases, that drives anti-Muslim prejudice.

## Introduction

Anti-Muslim prejudice is commonplace in Western democracies [1]. However, the vast majority of Muslims living in the West are peaceful [2]. Previous studies find that Muslim extremism dominates portrayals of Islam in the news [3, 4]. Because information about minority groups is typically indirect [5], critics argue that excessive media focus on radical Islam promotes fear and animosity toward all Muslims, not merely extremists [6]. Evidence for media-induced Islamophobia comes from experiments showing that news depictions of Islamic terrorism decreases tolerance of Muslims [7], and amplifies support for war against Muslim countries [8].

Contrary to the media-induced Islamophobia hypothesis, however, people tend to interpret the news in support of pre-existing beliefs [9, 10]. Indeed, people often seek affirmation from reporting that reinforces pre-existing opinions; people also tend to ignore or discount information that conflicts with their worldview [11, 12]. The literature on confirmatory biases suggests that media influences in tolerant societies will not increase prejudice; instead more basic demographic, educational, cultural, and political factors known to attenuate prejudice [13] might lead to a discounting of threatening generalizations about Muslims in response to media presentations of Islamic radicals. Finally, previous survey-based studies suggest that media-induced prejudice toward immigrants and ethnic minorities is restricted to areas where social tensions are high but does not extend to other regions [14, 15]. Given the absence of Muslim-specific threats across New Zealand, it would be unsurprising if there was no support for an association between media exposure and prejudice against Muslims.

To evaluate media-induced Islamophobia in the general population requires comparing responses to Muslims across a demographically and ideologically diverse non-Muslim population as they respond to the news. The hypothesis is best assessed in diverse and tolerant societies where there is where there is little intergroup competition for resources and few historical conflicts, and where anti-confirmatory biases might counterbalance one-sided presentations of Muslims as inherently threatening. Currently, however, national-scale quantitative studies of media-induced Islamophobia are lacking [16].

Here, we systematically investigate the media-induced Islamophobia hypothesis using a large and diverse national probability sample from one of the world’s most tolerant Western societies [17], where media coverage of Muslims is least likely to induce prejudice (*N* = 16,584, New Zealand). Muslims in New Zealand are ethnically and culturally diverse, and have peacefully co-existed for over 140 years [18]. The tolerant setting of New Zealand offers an especially strong challenge to the media-induced Islamophobia hypothesis because in the absence of intergroup conflict or competition, confirmatory biases can be expected to neutralize the effects of the media coverage that might lead to prejudicial generalizations about Muslims.

On the other hand, within New Zealand, tolerance of Muslims lags behind other minority groups, and it is unclear what, besides new coverage, could explain the Muslim acceptance gap. For example, 17.3% of New Zealanders report high levels of anger toward Muslims, whereas 9.1% report similar levels of anger toward Asians. S1 Table presents ratings of anger toward different minority groups among a sample of 14,022 non-Muslim, non-Arab and non-Asian New Zealanders. Muslims represent only 1.07% of the country’s population [19], and there is no history of violent intergroup conflict. Personal experiences with Muslims are thus unlikely to explain the Muslim acceptance gap.

### Modeling strategy

A key challenge to cross-sectional data is inferring causation. For example, increasing demand from those with pre-existing Muslim prejudice may create a demand for negative media representations of Muslims. Additionally, third variables, such as religious or regional influences may underpin observed correlations. Though inevitably limiting, we improve inference about media effects in five ways.

First, we simultaneously model attitudes toward Muslims, Arabs, and Asians as multivariate outcomes. The majority of Muslims in New Zealand are Asian (63.1%) whereas Arabs represent only 21% of New Zealand’s Muslims [19]. Nevertheless, depictions of Muslims in the New Zealand media are overwhelmingly Arab [3]. Because Arab Muslims are a small demographic group that is overrepresented in the news, a high correlation between Muslim and Arab prejudice combined with a relatively lower correlation between Muslim and Asian prejudice would be expected if media exposure were to fuel Islamophobia. By contrast, in the absence of media effects, such a correlation would remain puzzling [20]. We include attitudes toward these three groups in a single model to recover correlations in prejudice after adjusting for known demographic and ideological sources of prejudice, as well as for theoretically postulated media effects. We want to clarify that we do not test a theory of media-induced Asian or Arab prejudice, nor evaluate a theory of the relative size of association of media-exposure toward these groups. The advantages of our multivariate regression models are that they pool information about attitudes to the three minority groups within a single model to obtain more accurate estimates of all coefficients, while also obtaining more accurate estimates of prejudice correlations from the residuals of the models [21].

Second, our statistical models adjust for the co-variation of media exposure with demographic and ideological drivers of prejudice, which enables us to isolate the relative contribution of media influences over and above mechanisms identified in previous research [13].

Third, we adjust for correlated error terms arising from latent group-level dependencies by modelling religious denominations and geographical locations as random effects. This allows us to handle statistical non-independence arising from group-level clustering.

Fourth, we use Bayesian regression to estimate parameters in the model, which improves uncertainty estimates and affords transparent and intuitive probabilistic interpretations [22]. We use Bayesian estimation because we follow those who urge that statistical inference is best conceived as a process of estimating unknown, and in most cases unknowable, population parameters as best one can, with a clear appreciation of the inevitable limitations that confront inferences based on inherently uncertain magnitudes and systems [23].

Fifth, in both sets of analyses–(1) anger toward Muslims and (2) warmth toward Muslims—we model the interaction between political orientation and news exposure to estimate whether media exposure effects reliably differ among liberals and conservatives. Such differences would be expected if media effects were limited to mirroring pre-existing preferences among previously prejudiced segments of the population. By contrast, a news exposure–prejudice association that holds across the full political spectrum would be puzzling in the absence of media-fueled Islamophobia.

Though these five features of our modeling strategy improve inference, we emphasize that our method does not demonstrate causation. Rather, we believe the correlational evidence from our large and diverse Western sample helps to improve inference by affording a rigorous test of whether the predicted association between media exposure and Muslim prejudice holds after adjusting for all the other factors that might drive prejudice in a general population. However, our efforts to pragmatically improve inference should not be confused as a definitive test of media-induced Islamophobia hypothesis.

## Method

### Materials

The New Zealand Attitudes and Values Study (NZAVS) was approved by The University of Auckland Human Participants Ethics Committee on 03-June-2015 until 03-June-2018 (reference Number: 014889). The NZAVS is an annual, longitudinal national probability panel of registered New Zealand voters, which was started in 2009. We analyzed data from participants who completed the Time 5 wave of the NZAVS, the most recent wave for which data was entered at the time of this study, and the first wave with measures of news exposure. The Time 5 (2013) wave of the NZAVS contained responses from 18,261 participants (10,502 retained from one or more previous waves, 7,581 new additions from booster sampling, and 181 unmatched participants or unsolicited opt-ins). The sample retained 3,934 participants from the initial Time 1 (2009) NZAVS of 6,518 participants (a retention rate of 60.4% over four years), and 9,844 participants from the full Time 4 (2012) sample (a retention rate of 80.8% from the previous year). Participants were mailed a copy of the questionnaire, with a second postal follow-up two months later. Participants who provided an email address were also emailed and invited to complete an on-line version if they preferred. We offered a prize draw for participation, non-respondents were emailed and phoned multiple times, and all participants were mailed a Season’s Greetings card from the NZAVS research team which informed them that they had been automatically entered into a bonus seasonal grocery voucher prize draw. We also mailed our yearly pamphlet summarizing key research findings published during the most recent wave of the study.

### Participants

The Time 5 (2013) wave of the NZAVS included 18,261 respondents. Of these participants, 40 self-identified as Muslim, 30 as Middle Eastern, and 796 others as Asian. Because we were interested in out-group determinants of prejudice toward Arabs, Asians, and Muslims, only non-Muslim, non-Middle Eastern born, non-Asian participants were included in the analyses. Moreover, an additional 811 participants did not indicate how often they attend to the news, and were removed from analyses. This resulted in a sample of *N* = 16,584. Missing data for demographic variables, religious identification, and political orientation were multiply imputed using the Amelia package in R [24]. Missing data frequencies were relatively low across responses to most variables, with missingness typically observed at less than 2%. An exception was in responses to education, where missing responses were observed for 10.6% of the participants.

### Missing data

We adopted two strategies for handling missing data. First, we modeled associations using multiple imputation, in which missing responses were imputed using the Amelia package in R [24]. Multiple imputation of missing data preserves information and attenuates the effects of response biases in conditions where the causes of missingness may be predicted from other observed variables. For this reason, we prefer multiple imputation, and report multiply-imputed datasets in the main text of this study. However, to assess whether this choice affected our results, a second identical series of analyses were conducted using a dataset in which participants with missing fixed effects responses were deleted pairwise, resulting in a sample of *N* = 14,022. There were no practically important differences in the interpretation of results for the two methods of handling missing data. The multiple imputation strategy we favor shrunk coefficients estimates closer to zero, and is therefore more conservative. Sample information and results for the analysis using pairwise deletion are reported in S2—S10 Tables. Additionally, a side-by-side comparison of the solutions using (1) the multiply imputed dataset and (2) the pairwise deleted dataset are presented in S1 and S2 Figs.

For multiple imputation, the following yes/no indicators (0 = “No”, 1 = “Yes”) were entered as nominal responses (factors): European Ethnicity, male gender, employment status, parental status, partner status, and urban location. The remaining missing indicators for fixed effects predictors were assumed to be continuous real numbers. Indicators for denominations (a factor with 85 levels) and territorial authorities (a factor with 67 levels) were not imputed by Amelia because random effects can be imputed during Markov Chain Monte Carlo (MCMC) estimation [25]. Additionally, MCMCglmm imputes missing outcome responses during MCMC, hence the multivariate outcomes in the first analysis: “Anger To Arabs”, and “Anger to Immigrants,” and “Anger to Muslims,” and in the second analysis: “Warmth To Arabs”, and “Warmth to Immigrants,” and “Warmth to Muslims” were not imputed using Amelia but rather estimated during MCMC [25]. The posterior distributions that are generated from MCMC are probabilistic distributions for modeled associations, which are conditional on the data, model, and priors (note: owing to the size and diversity of the dataset we use non-informative priors).

Following Amelia package recommendations, where low frequencies of missing responses are observed, we imputed five missing datasets. Multivariate multilevel regression models were performed separately on each dataset, and the MCMC chains were subsequently combined to obtain aggregate estimates, because in each analysis the models were (conditionally) independent of the others [18]. To reiterate, although estimates varied somewhat between the imputed datasets and the pairwise-deleted dataset, key theoretical inferences were identical. In other words, for this study, it made no practical difference whether multiply imputed or pairwise deleted datasets were used. We report the results of multiply imputed datasets in the main text because multiply imputed datasets lead to better population level inferences [26].

### Measures

Table 1 reports the sample means, standard deviations, and response ranges for the data.

**Table 1:** A summary of all variables used in analyses (*N* = 16,548), including means, standard deviations, and ranges.

Variable	Mean	SD	Min	Max
Anger toward Arabs	2.85	1.75	1	7
Anger toward Asians	2.55	1.59	1	7
Anger toward Muslims	2.93	1.83	1	7
Warmth toward Arabs	3.80	1.50	1	7
Warmth toward Asians	4.50	1.32	1	7
Warmth toward Muslims	3.75	1.56	1	7
Hours of news	5.21	5.19	0	120
Political Conservatism	3.64	1.29	1	7
Religious Identification	1.80	2.61	0	7
Age	47.97	13.99	18	94
Education	5.92	2.84	0	10
Employed	0.76	0.42	0	1
European ancestry	0.94	0.25	0	1
Gender	0.37	0.48	0	1
Socioeconomic deprivation	4.78	2.78	1	10
Parent	0.74	0.44	0	1
Partner	0.74	0.44	0	1
Urban dwelling	0.67	0.47	0	1

#### Anger

Attitudes toward minority groups were assessed using two separate feeling-thermometer ratings, (1) anger and (2) warmth [25]. Anger toward Arabs, Asians and Muslims was assessed by asking participants to indicate how angry they feel toward each group on a scale of 1–7 where 1 indicated “no anger”, 4 indicated “neutral,” and 7 indicated “anger” (Arabs: *M* = 2.85, *SD* = 1.75; Asians: *M* = 2.55, *SD* = 1.59; Muslims: *M* = 2.93, *SD* = 1.83).

#### Warmth

Warmth toward Arabs, Asians and Muslims was assessed by asking participants to indicate how warm they feel toward each group on a scale of 1–7 where 1 indicated “least warm”, 4 indicated “neutral,” and 7 indicated “most warm” (Arabs: *M* = 3.84, *SD* = 1.50; Asians: *M* = 4.50, *SD* = 1.32; Muslims: *M* = 3.72, *SD* = 1.56).

#### Hours of news

Hours of news exposure was assessed by asking participants how many hours they watched or read about the news in the past week (*M* = 5.21, *SD* = 5.19). S3 to S5 Figs plot raw hours of news exposure and anger toward each of the three outcome variables, and S6 to S8 Figs plot raw hours of news exposure and warmth toward each of the three outcome variables. S9 to S11 Figs plot log hours of news exposure and anger toward each of the three outcome variables, and S12 to S14 Figs plot log hours of news exposure and warmth toward each of the three outcome variables.

#### Political conservatism

Political conservatism was assessed using a single-item that asked participants to report their political orientation on a 1 (Liberal) to 7 (Conservative) scale (*M* = 3.63, *SD* = 1.29).

#### Religious identification

To assess religious identification, we asked people: “Do you identify with a religion and/or spiritual group?”. For those who identified with a religion/spiritual group, we asked participants to rate on a scale from 1–7 “how important is your religion/spiritual group to how you see yourself?” Those individuals who indicated that they did not belong to a religion were coded as a 0 (*n* = 10,129) on this scale (*M* = 1.84; *SD* = 2.61).

#### Age

The mean age of the sample was 47.97 (*SD* = 13.99).

#### Education

Education was coded as either no qualification “0” (*n* = 748), Level 1 Certificate “1” (*n* = 2,195), Level 2 Certificate “2” (*n* = 1,037), Level 3 Certificate “3” (*n* = 1,901), Level 4 Certificate “4” (*n* = 862), Level 5 Diploma/Certificate “5” (*n* = 1,279), Level 6 Graduate Certificate/Diploma “6” (*n* = 773), Bachelor’s Degree/Level 7 Diploma/Certificate “7” (*n* = 3,725), Postgraduate Diploma/Certificate “8” (*n* = 1,402), Master’s Degree “9” (*n* = 1,054), or Doctorate Degree “10” (*n* = 296).

#### Employment

Employment status was assessed by asking participants if they were currently working. “Yes” was coded as “1” (*n* = 12,585) and “no” was coded as “0” (*n* = 3,883).

#### European ancestry

We also assessed ethnic origin, and 15,516 participants indicated that they were of European descent (coded as 1), whereas 1,068 indicated non-European ancestry (coded as 0).

#### Gender

The sample included 6,190 males (coded as 1) and 10,391 females (coded as 0).

#### Socioeconomic deprivation

We measured the socio-economic status of participants’ immediate (small area) neighborhood using the 2013 New Zealand Deprivation Index [27]. New Zealand is unusual in having rich census information about each area unit/neighborhood of the country that is made available for research purposes. The smallest of these area units are meshblocks. The NZAVS includes the meshblock code for each participant.

The geographic size of meshblock units differs depending on population density. Each unit tends to cover a region containing a median of roughly 81 residents (*M* = 95.95, *SD* = 73.49, range = 0 to 1899). In 2013, there were a total of 44,211 meshblocks for which data were available. The New Zealand census defines a meshblock as, “a defined geographic area, varying in size from part of a city block to large areas of rural land”. Each meshblock abuts against another to form a network covering all of New Zealand including coasts and inlets, and extending out to the two-hundred-mile economic zone. Meshblocks are added together to build up larger geographic areas such as area units and urban areas.

The New Zealand Deprivation Index uses aggregate census information about the residents of each meshblock to assign a decile-rank index from 1 (most affluent) to 10 (most impoverished) to each meshblock unit [27]. Because it is a decile-ranked index, the 10% of meshblocks that are most affluent are given a score of 1, the next 10% a score of 2, and so on. The index is based on a Principal Components Analysis of the following nine variables (in weighted order): proportion of adults who received a means-tested benefit, household income, proportion not owning own home, proportion single-parent families, proportion unemployed, proportion lacking qualifications, proportion household crowding, proportion no telephone access, and proportion no car access.

The New Zealand Deprivation Index thus reflects the average level of deprivation for small neighborhood-type units (or small community areas of about 80–90 people each) across the entire country. The measure is a well-validated index of the level of deprivation of small area units, and has been widely used in health and social policy research examining numerous health outcomes. Our sample had a mean deprivation index of *M* = 4.78 (*SD* = 2.78).

#### Parental status

We assessed parental status by asking participants to indicate their number of children. Participants were coded as “0” if they reported that they do not have children (*n* = 4,331) and “1” if they reported that they do (*n* = 12,253).

#### Partner

Participants were asked if they were in a relationship. “Yes” was coded as “1” (*n* = 11,976) and “no” was coded as “0” (*n* = 4,533).

#### Urban dwelling

People were coded as either residing in an urban “1” (*n* = 10,856) or rural “0” (*n* = 5,581) area based on New Zealand census data.

#### Baseline population for reported expected values

Expected values for model estimates were obtained to clarify the magnitude of expected changes in anger and warmth in response to news exposure. For computational reasons, MCMCglmm required us to pass the average of news coverage in the complete case sub-sample, which will have slightly underestimated the news exposure effect. This is because currently MCMCglmm’s predictions functions cannot handle multiple datasets. Specifically, we used a mean 5.13 and SD of 5.23 (i.e., pairwise deleted means) hours per week instead of 5.21 and 5.19 (i.e., imputed means). Hence the reported estimates are slightly more conservative. We repeat that the pairwise deleted dataset leads to an identical inference. We report these modeling decisions so that others can better replicate our findings.

The baseline population for inferred expected values from news exposure is as follows: urban male New Zealanders of European ancestry who were employed, single, and had no children, had average levels of education, no religious identification, average political conservatism, and average socioeconomic deprivation. 

#### Statistical analysis

Statistical analysis was performed using R version 3.2.4 (2016-03-10) [28] on an Apple Macbook Pro Platform: x86_64-apple-darwin13.4.0 (64-bit), running under: OS X 10.11.4 (El Capitan). Bayesian multivariate, multilevel regression analysis was performed using MCMCglmm version 2.18.2 [25, 21]. In addition to MCMCglmm [25], we used the following R packages: Amelia [24], coefplot2 [29], ggplot2 [30], GridExtra [31], and their dependencies, Ape [32], coda [33], Rcpp [34], Lattice [35], Matrix [36], and Seamless [34]. Priors for the effects modeled as fixed in the current study were uninformative, with a mean of zero and variance of 10^8^ [21]. Following Hadfield, we used parameter-expanded priors to adjust for the variance of denominations and territorial authorities [21]. Parameter-expanded priors were centered at zero, and assumed a variance of 10^2^, which on this dataset were not informative [21]. Priors on residual covariance matrices were centered at zero, and assigned a non-informative normal inverse Wishart distribution, with a shape of 3 and scale of .002. Hence, solutions for variance and covariance components were informed by the data and not the priors, which is appropriate given the size of the dataset and empirical uncertainty about the hypothesis tested in this study.

To facilitate interpretation and MCMC mixing, religious identification, political conservatism and socioeconomic deprivation were centered at their respective means and standardized. Regression model intercept terms are interpretable as the expected outcomes when predictors are set to zero, in this case: the expected level of anger in the population of respondents who are average sample age (47.92 years old), high school educated, do not identify as European, not-religiously identified, female, of average political conservatism, without any partner, without any children, of average socio-economic deprivation, and living in a rural setting.

To adjust for multi-level dependencies, we modeled denominational and geographical region intercepts as random-effects. Intercept variances were centered at zero, and variance/co-variance coefficients were estimated for denominations and geographic regions. Denominations were classified using the 2013 New Zealand census categories, which contains 93 categories, of which 85 were represented in our response profiles [18]. Territorial authorities are geographic units defined by the New Zealand Census, under the Local Government Act 2002. There are currently 67 territorial authorities in New Zealand consisting of 14 city councils and 53 districts.

The default number of MCMC cycles in MCMCglmm is 13,000, with a burn-in of 3,000 cycles and a thin interval of 10. We ran models for 103,000 cycles with a burnin of 3,000 cycles and a thin interval of 10. Models were run separately on each of the five multiply imputed data sets. Evidence from plots and the MCMCglmm autocorr() function of the posterior distributions for all models indicated MCMC chains mixed well and there was no evidence of auto-correlation. The chains, which were (conditionally) independent, were combined, and the posterior distributions for inferred coefficients reported were the average results.

## Results

**Fig 1:**
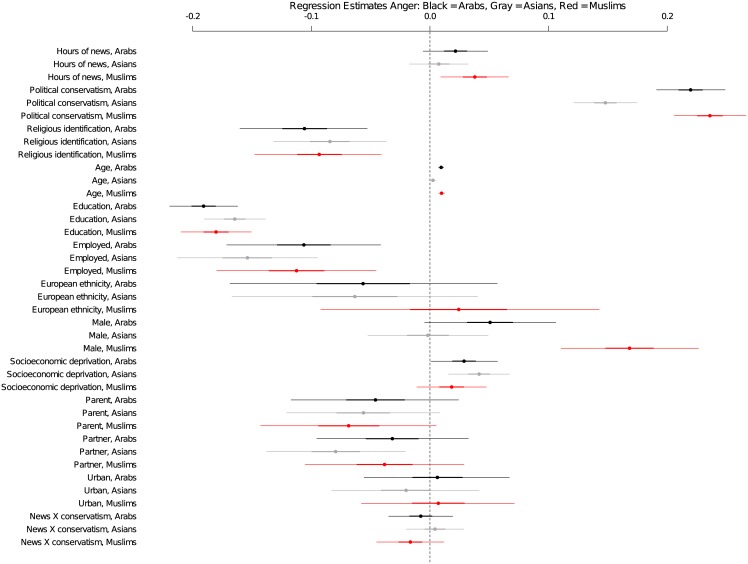
Coefficients of predictors for anger toward Muslims. A graph of the posterior means and 95% posterior density intervals for all anger coefficients in the model, allowing a relative comparison of news effects with known drivers of minority group prejudice.

In the analysis focusing on anger, we find strong evidence that news exposure is associated with greater anger toward Muslims [b = 0.038; HPD Interval: 0.009, 0.066; pMCMC = 0.009]. Though support for the effects of exposure on anger toward Arabs is more variable than the effect of news exposure on anger toward Muslims, anger toward Arabs trends in the same direction as anger toward Muslims [b = 0.021; HPD Interval: -0.006, 0.049; pMCMC = 0.122; Fig 1 & Table 2]. By contrast, we find no support for an association between news exposure and anger toward Asians, the majority ethnic group of Muslims in New Zealand [b = 0.008; HPD Interval: -0.017, 0.032; pMCMC = 0.558]. Additionally, we find no support for reliable differences among liberals and conservatives in anger toward Muslims (or any other group) from news exposure [b = -0.016; HPD Interval: -0.045, 0.012; pMCMC < 0.252]. Rather, the positive relationship between greater media exposure and greater anger toward Muslims is consistent across the political spectrum. Table 2 and Fig 1 present the posterior means and 95% posterior density intervals for all anger coefficients in the model. Additional information on this model is presented in S11—S13 Tables.

**Table 2:** Results of a Bayesian regression model (*N* = 16,548) predicting anger toward Arabs, Asians, and Muslims, with 95% posterior density intervals. Political conservatism, religious identification, and socioeconomic deprivation were standardized, and age and education were centered.

	_Anger toward Arabs_			_Anger toward Asians_			_Anger toward Muslims_		
	_Posterior Mean_	_95% Lower Bounds_	_95% Upper Bounds_	_Posterior Mean_	_95% Lower Bounds_	_95% Upper Bounds_	_Posterior Mean_	_95% Lower Bounds_	_95% Upper Bounds_
_**Intercept**_	3.079	2.910	3.248	2.871	2.721	3.020	3.050	2.885	3.219
_**Hours of news**_	0.021	-0.006	0.049	0.008	-0.017	0.032	0.038	0.009	0.066
_**Political conservatism (standardized)**_	0.220	0.191	0.249	0.148	0.121	0.174	0.236	0.206	0.266
_**Religious identification (standardized)**_	-0.106	-0.160	-0.053	-0.084	-0.132	-0.037	-0.093	-0.148	-0.041
_**Age (centered)**_	0.009	0.007	0.012	0.003	0.000	0.005	0.010	0.007	0.012
_**Education (centered)**_	-0.191	-0.219	-0.162	-0.165	-0.190	-0.139	-0.180	-0.210	-0.151
_**Employed**_	-0.106	-0.171	-0.042	-0.154	-0.213	-0.095	-0.112	-0.180	-0.045
_**European**_	-0.056	-0.169	0.057	-0.063	-0.167	0.040	0.024	-0.092	0.143
_**Gender**_	0.051	-0.004	0.106	-0.002	-0.052	0.049	0.168	0.110	0.226
_**Socioeconomic deprivation (standardized)**_	0.029	0.001	0.057	0.041	0.016	0.067	0.018	-0.011	0.048
_**Parent**_	-0.046	-0.117	0.024	-0.056	-0.121	0.008	-0.068	-0.143	0.005
_**Partner**_	-0.032	-0.095	0.032	-0.080	-0.138	-0.021	-0.038	-0.105	0.029
_**Urban**_	0.006	-0.055	0.067	-0.020	-0.083	0.041	0.007	-0.058	0.071
_**Hours of news X Political conservatism**_	-0.008	-0.035	0.019	0.004	-0.020	0.029	-0.016	-0.045	0.012

**Fig 2:**
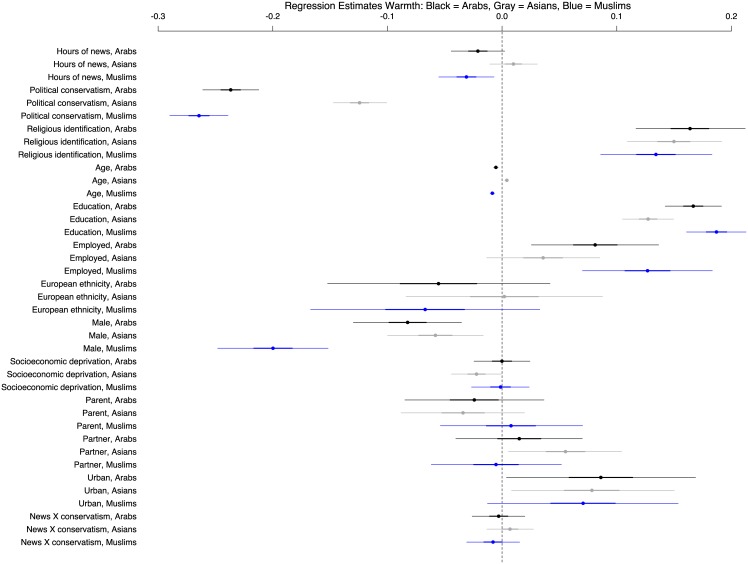
Coefficients of predictors for warmth toward Muslims. A graph of the posterior means and 95% posterior density intervals for all warmth coefficients in the model, allowing a relative comparison of news effects with known drivers of minority group prejudice.

The second series of analyses, which focused on warmth, exhibits a similar pattern to the anger analysis. News exposure is strongly associated with reduced warmth toward Muslims [b = -0.031 HPD Interval: -0.055, -0.007; pMCMC = 0.011]. Here again, the association between media exposure and warmth toward Arabs trends in the same direction as the association between media exposure and warmth towards Muslims, but is more variable [b = -0.021; HPD Interval: -0.044, 0.002; pMCMC = 0.077]. By contrast, we find no support for a reliable association between news exposure and warmth toward Asians, the majority ethnic group of Muslims in New Zealand [b = 0.010; HPD Interval: -0.011, 0.031; pMCMC = 0.350; Fig 2 and Table 3]. Additionally, there is no support for reliable differences in warmth toward Muslims (or any other group) among liberals and conservatives from news exposure [b = -0.008; HPD Interval: -0.031, 0.015; pMCMC = 0.503]. As with the anger responses, the clear linear relationship between greater media exposure and reduced warmth toward Muslims is consistent across the political spectrum. Table 3 and Fig 2 present the posterior means and 95% posterior density intervals for all warmth coefficients in the model. Additional information about this model is presented in S14—S16 Tables.

**Table 3:** Results of a Bayesian regression model (*N* = 16,548) predicting warmth toward Arabs, Asians, and Muslims, with 95% posterior density intervals. Political conservatism, religious identification, and socioeconomic deprivation were standardized, and age and education were centered.

	_Warmth toward Arabs_			_Warmth toward Asians_			_Acceptance of Muslims_		
	_Posterior Mean_	_95% Lower Bounds_	_95% Upper Bounds_	_Posterior Mean_	_95% Lower Bounds_	_95% Upper Bounds_	_Posterior Mean_	_95% Lower Bounds_	_95% Upper Bounds_
_**Intercept**_	3.712	3.568	3.854	4.377	4.251	4.500	3.706	3.558	3.850
_**Hours of news**_	-0.021	-0.044	0.002	0.010	-0.011	0.031	-0.031	-0.055	-0.007
_**Political conservatism (standardized)**_	-0.237	-0.261	-0.212	-0.124	-0.147	-0.101	-0.264	-0.290	-0.239
_**Religious identification (standardized)**_	0.164	0.117	0.212	0.15	0.109	0.191	0.134	0.086	0.183
_**Age (centered)**_	-0.005	-0.007	-0.003	0.004	0.002	0.006	-0.008	-0.010	-0.007
_**Education (centered)**_	0.167	0.142	0.191	0.127	0.105	0.150	0.187	0.161	0.213
_**Employed**_	0.081	0.026	0.137	0.036	-0.014	0.085	0.127	0.070	0.183
_**European**_	-0.055	-0.152	0.042	0.002	-0.084	0.088	-0.067	-0.167	0.033
_**Gender**_	-0.082	-0.13	-0.035	-0.058	-0.100	-0.016	-0.200	-0.248	-0.152
_**Socioeconomic deprivation (standardized)**_	0.000	-0.024	0.024	-0.022	-0.044	-0.001	-0.001	-0.027	0.024
_**Parent**_	-0.024	-0.085	0.037	-0.034	-0.088	0.020	0.008	-0.054	0.070
_**Partner**_	0.015	-0.04	0.070	0.055	0.006	0.104	-0.005	-0.062	0.052
_**Urban**_	0.086	0.004	0.169	0.078	0.008	0.150	0.071	-0.013	0.154
_**Hours of news X Political conservatism**_	-0.003	-0.026	0.020	0.007	-0.013	0.027	-0.008	-0.031	0.015

Because we simultaneously model attitudes to Arabs, Asians, and Muslims, we are able to quantify prejudices toward different groups. S13 Table presents the variance/covariance estimates for anger and S16 Table presents the variance/covariance estimates for warmth. S11, S12, S14 and S15 Tables present the variance/covariance estimates for denominational and regional dependences in anger and warmth responses. The correlation between anger toward Muslims and anger toward Arabs is 0.822 and for warmth toward Muslims and warmth toward Arabs it is 0.817. By contrast, the correlation between anger toward Arabs and anger toward Asians is 0.734; the correlation between warmth toward Arabs and warmth toward Asians is 0.627; the correlation between anger toward Asians and anger toward Muslims is 0.647; the correlation between warmth toward Asians and warmth toward Muslims is 0.580. New Zealand Muslims of Asian descent are over three times more numerous than New Zealand’s Muslims of Arab descent; however, anti-Muslim and anti-Arab prejudice are more strongly correlated with each other than either was with anti-Asian prejudice. This provides additional evidence that Islam and Arab ethnicity are conflated among the general population.

Examining the links between news exposure and Muslim prejudice allows quantification of the magnitude of media-induced Islamophobia. Expected anger (1 = no anger; 7 = anger) toward Muslims among those who do not attend to the news is 3.099 [2.657, 3.546]. At the sample mean of 5.235 hours of news exposure per week, expected anger is 3.138 [2.666, 3.614]; a one standard deviation increase in news exposure (10.360 per week) predicts average anger toward Muslims at 3.176 [2.675, 3.680]; a two standard deviation increase in news exposure (15.486 hours per week) predicts average anger toward Muslims at 3.213 [2.684, 3.7463].

Turning to warmth (1 = least warm; 7 = most warm), among those who do not attend to the news, expected average warmth is 3.668 [3.256, 4.075]. At the sample mean of 5.235 hours per week, expected average warmth drops to 3.636 [3.200, 4.068]; a one standard deviation increase in news exposure (10.470 hours per week) predicts average warmth toward Muslims at 3.605 [3.145, 4.061], a two standard deviation shift in news exposure (15.486 hours per week) predicts average warmth toward Muslims at 3.574 [3.090, 4.054].

**Fig 3:**
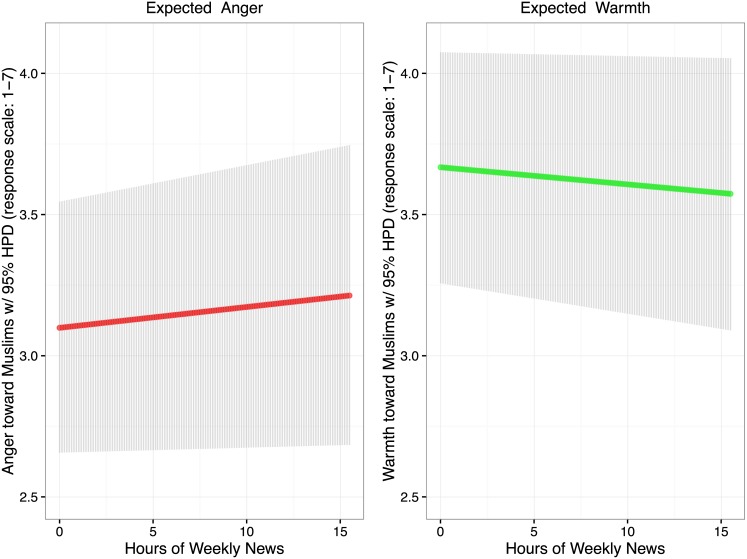
Expected anger toward Muslims and expected warmth toward Muslims in response to news exposure across two standard deviations of new exposure (0–15.486 hours).

How large is the effect of news exposure? We can compare the expected effect of news exposure on anger toward Muslims to the increase in expected anger across two standard deviations of the political spectrum. Fig 3 graphs such expectations with their 95% prediction intervals. Among people who do not watch the news, expected anger is 3.099 [2.657, 3.546]; a one standard deviation shift right in political conservatism is expected to increase average anger to 3.352 [2.909, 3.801] and a two standard deviation is expected to increase average anger to 3.605 [3.160, 4.055]. The magnitude of the expected news exposure on anger to Muslims is 30.5% as great as that of political orientation.

**Fig 4:**
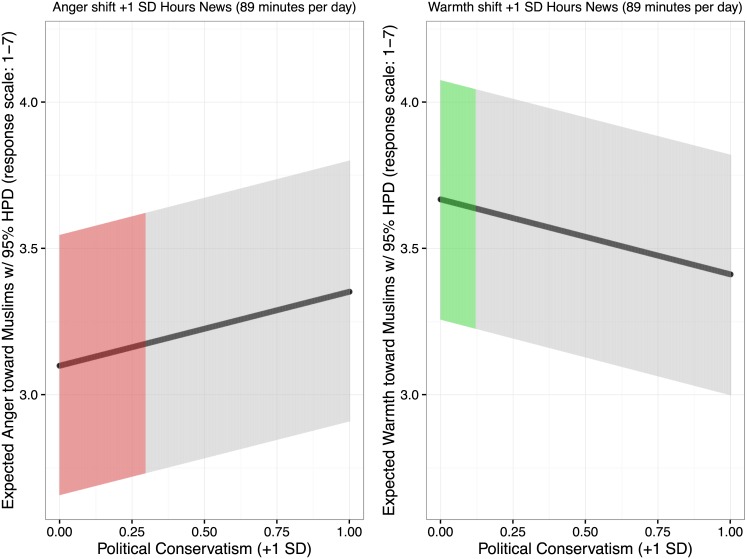
Expected effects of news exposure on anger (red) and warmth (green) toward Muslims compared to the expected effects of political orientation on anger and warmth toward Muslims. The response scale for news exposure and for conservatism is one standard deviation. In standard deviation units, the expected news exposure effect for anger is 30.5% that of the political orientation effect; the expected news exposure effect for warmth is 12.5% that of the political orientation effect.

Turning to warmth (1 = least warm; 7 = most warm), among people who do not watch the news, expected warmth is 3.668 [3.256, 4.075]; a one standard deviation shift right in political conservatism is expected to decrease average warmth to 3.411 [3.820, 2.998], and a two standard deviation shift in conservatism is expected to decrease average warmth to 3.155 [3.566, 2.740]. The magnitude of the expected news exposure response on warmth toward Muslims is 12.4% as great as that of political orientation. Fig 4 compares expected anger and expected warmth in response to news exposure to the strongest predictor of anger and warmth toward Muslims, political orientation.

## Discussion

The present study is interesting both for its methods and findings.

### Importance of methods

Though our analyses are based on cross-sectional survey data, our modeling strategy improved inference in five ways. First, we simultaneously modeled attitudes toward Muslims, Arabs, and Asians. We find that increased news exposure is related to increased anti-Muslim prejudice, a trend toward increased Arab prejudice, and no reliable evidence for an effect of news exposure on attitudes toward Asians. Because most Muslims in New Zealand are Asian, these results would be puzzling in the absence of media-driven Islamophobia [37]. Second, we improved inference by including known drivers of minority group prejudice in our models. We find that even after adjusting for these predictors, there remained evidence for a media influenced Islamophobia effect. Third, we modeled both religious denominations and geographic area as random effects, which allowed us to adjust for correlated error terms arising from these group level dependencies. Fourth, we used Bayesian regression to estimate parameters in the model, which efficiently pooled uncertainty and afforded transparent and intuitive probabilistic interpretation. Finally, we assessed the interaction of news exposure and political orientation which allowed us to investigate whether media effects differed among liberals and conservatives. An association between increased news exposure and increased anti-Muslim prejudice across the political spectrum would be puzzling in the absence of media-fueled Islamophobia. The relationship we observed reveals a pattern that is consistent across the political spectrum. Results indicate that news exposure is associated with Muslim prejudice among liberals as well as among conservatives.

### Importance of results

Our study is among the first to systematically investigate media-induced Islamophobia at a national scale. We emphasize that our study is based on cross-sectional data and does not establish causation. What we find is a reliable linear association between media exposure and Muslim prejudice in a peaceful and tolerant society, with no history of regional Muslim conflicts. In 2013, people in the general New Zealand population who were exposed more to the news were expected to exhibit greater anger toward Muslims, and lower warmth towards Muslims. Our study did not attempt to compare media effects for prejudice on different groups, and we do not infer that news exposure and minority group prejudice is stronger for one group compared to the others. Allport’s general question of how media exposure affects attitudes to minority groups remains a fruitful horizon for future research [5]. However, we do not find a reliable association between media exposure and tolerance of Asians, the majority ethnicity of Muslims in New Zealand. Rather, we find that the association between news exposure and prejudice is specific to Muslims (and less reliably to Arabs), and is linear across the political spectrum. These results are consistent with a theory that predicts media exposure increases Muslim prejudice. Moreover, these results are inconsistent with an alternative theory that predicts generally prejudiced people seek greater news exposure. Hence, absent media effects, these results do not readily lend themselves to plausible theoretical alternatives at present. We want to underscore that our results do not definitively exclude alternative theories, and we hope the present study stimulates future national-scale research about the causes and consequences of Muslim prejudice.

What is the practical interest of our study? Most Muslim organizations advocate religious tolerance and peace [38]. The relationship between media exposure and Muslim prejudice implies the media are not conveying this larger picture. Speculating about why media exposure is reliably associated with anti-Muslim prejudice, we notice that previous research finds that stories evoking fear and anger garner more space and more prominent positions in print and visual media [39]. Notably, previous studies on the content of New Zealand media find that representations of Muslims are overwhelmingly negative [3]. The data used in our analyses were collected in 2013, a year in which nearly 10,000 terrorist attacks occurred, an increase of 44% from the previous year [40]. Sixty-six percent of terrorist attacks in 2013 were performed by groups who claimed an extreme interpretation of Islam [40]. On average, those who attended to the news more frequently would have experienced more exposure to Muslim extremism. Why do the media present threatening images of Muslims? Previous research indicates that people are motivated to learn about social threats [39]. When making social evaluations, however, people tend to give more credence to negative information [41]. Researchers argue that profiling violence owes to media competition [42]. Importantly, it has been found that threatening media representations inflate perceptions of social risks [43]. Worryingly, the frequency of exposure to violence is more predictive of social attitudes than recent non-negative exposure [44], implying that any media fuelled amplification of prejudice toward Muslims may have long-term consequences.

Recent coverage of radical Muslim terrorism has renewed controversies about the media’s role in inflaming prejudice against Muslims. Our demonstration of a clear association between media exposure and anti-Muslim prejudice, which applies across the political spectrum, lends support to worries that media reporting—at least in 2013 –was tipping the scale toward anger. Media induced Islamophobia is all the more concerning if Islamic extremists are attacking Western targets to maximize the effects of media induced anger [6]. At the very least, prejudice is known to inflict substantial personal and social harms [45].

However, concerns about the media’s propensity for provoking social harm are hardly new. Two centuries ago James Madison wrote, “[s]ome degree of abuse is inseparable from the proper use of everything, and in no instance is this more true than in that of the press” [46]. In weighing up the costs of censorship against the benefits of information, the architects of modern democracies sided with openness. It is important to remember that the media are not fated to sell stories that exploit majority group fears, and that much media reporting of Muslims is not inflammatory. Indeed, reporting that humanizes minority groups may increase acceptance [15]. To increase Muslim acceptance in Western democracies may require a broader exercise of journalistic freedoms, not less.

## Supporting information

S1 Appendix Summary of measures, pairwise deleted dataset. (DOCX)

S1 Table Absolute levels of anger toward Asians and Muslims on a scale from 1–7. (DOCX)

S2 Table A summary of all variables used in analyses of the pairwise deleted dataset (*N* = 14,022), including means, standard deviations, and ranges. (DOCX)

S3 Table Variance and covariance solutions for religious denominations (n = 93) of a Bayesian regression model of the pairwise deleted dataset (*N* = 14,022) predicting anger toward Arabs, Asians, and Muslims. (DOCX)

S4 Table Variance and covariance solutions for geographic regions (n = 67) of a Bayesian regression model of the pairwise deleted dataset (*N* = 14,022) predicting anger toward Arabs, Asians, and Muslims. (DOCX)

S5 Table Residual variance structure of a Bayesian regression model of the pairwise deleted dataset (*N* = 14,022) predicting anger toward Arabs, Asians, and Muslims. (DOCX)

S6 Table Results of a Bayesian regression model of the pairwise deleted dataset (*N* = 14,022) predicting anger toward Arabs, Asians, and Muslims. Political conservatism, religious identification, and socioeconomic deprivation were standardized, and age and education were centered.
(DOCX)

S7 Table Variance and covariance solutions for religious denominations (n = 93) of a Bayesian regression model of the pairwise deleted dataset (*N* = 14,022) predicting warmth toward Arabs, Asians, and Muslims. (DOCX)

S8 Table Variance and covariance solutions for geographic regions (n = 67) of a Bayesian regression model of the pairwise deleted dataset (*N* = 14,022) predicting warmth toward Arabs, Asians, and Muslims. (DOCX)

S9 Table Residual variance structure of a Bayesian regression model of the pairwise deleted dataset (*N* = 14,022) predicting warmth toward Arabs, Asians, and Muslims. (DOCX)

S10 Table Results of a Bayesian regression model of the pairwise deleted dataset (*N* = 14,022) predicting warmth toward Arabs, Asians, and Muslims. Political conservatism, religious identification, and socioeconomic deprivation were standardized, and age and education were centered.
(DOCX)

S11 Table Variance and covariance solutions for religious denominations (n = 93) of a Bayesian regression model of the Ameila imputed dataset (*N* = 16,548) predicting anger toward Arabs, Asians, and Muslims. (DOCX)

S12 Table Variance and covariance solutions for geographic regions (n = 67) of a Bayesian regression model of the Ameila imputed dataset (*N* = 16,548) predicting anger toward Arabs, Asians, and Muslims. (DOCX)

S13 Table Residual variance structure of a Bayesian regression model of the Ameila imputed dataset (*N* = 16,548) predicting anger toward Arabs, Asians, and Muslims. (DOCX)

S14 Table Variance and covariance solutions for religious denominations (n = 93) of a Bayesian regression model of the Ameila imputed dataset (*N* = 16,548) predicting warmth toward Arabs, Asians, and Muslims. (DOCX)

S15 Table Variance and covariance solutions for geographic regions (n = 67) of a Bayesian regression model of the Ameila imputed dataset (*N* = 16,548) predicting warmth toward Arabs, Asians, and Muslims. (DOCX)

S16 Table Residual variance structure of a Bayesian regression model of the Ameila imputed dataset (*N* = 16,548) predicting warmth toward Arabs, Asians, and Muslims. (DOCX)

S1 Fig Comparison of coefficients of predictors of anger toward Muslims on imputed and pairwise deleted datasets. (EPS)

S2 Fig Comparison of coefficients of predictors of acceptance of Muslims on imputed and pairwise deleted datasets. (EPS)

S3 Fig Scatterplot of weekly hours of news and anger toward Arabs. (EPS)

S4 Fig Scatterplot of weekly hours of news and anger toward Asians. (EPS)

S5 Fig Scatterplot of weekly hours of news and anger toward Muslims. (EPS)

S6 Fig Scatterplot of weekly hours of news and warmth toward Arabs. (EPS)

S7 Fig Scatterplot of weekly hours of news and warmth toward Asians. (EPS)

S8 Fig Scatterplot of weekly hours of news and warmth toward Muslims. (EPS)

S9 Fig Scatterplot of log weekly hours of news and anger toward Arabs. (EPS)

S10 Fig Scatterplot of log weekly hours of news and anger toward Asians. (EPS)

S11 Fig Scatterplot of log weekly hours of news and anger toward Muslims. (EPS)

S12 Fig Scatterplot of log weekly hours of news and warmth toward Arabs. (EPS)

S13 Fig Scatterplot of log weekly hours of news and warmth toward Asians. (EPS)

S14 Fig Scatterplot of log weekly hours of news and warmth toward Muslims. (EPS)
