# Supplementary material for: News exposure predicts anti-Muslim prejudice
Source: PLoS One. 2017 Mar 31;12(3):e0174606. doi: 10.1371/journal.pone.0174606 (PMC5375159; doi:10.1371/journal.pone.0174606)
Supplement: S1 Appendix — (DOCX) [file pone.0174606.s001.docx]

**S1 Appendix.** Summary of Measures, Pairwise Deleted Dataset.

**Anger** Anger toward Arabs, Asians and Muslims was assessed by asking participants to indicate how angry they feel toward each group on a scale of 1-7 where 1 indicated “no anger”, 4 indicated “neutral,” and 7 indicated “anger” (Arabs: *M* = 2.82, *SD* = 1.73; Asians: *M* = 2.51, *SD* = 1.57; Muslims: *M* = 2.89, *SD* = 1.81).

**Warmth** Warmth toward Arabs, Asians and Muslims was assessed by asking participants to indicate how warm they feel toward each group on a scale of 1-7 where 1 indicated “least warm”, 4 indicated “neutral,” and 7 indicated “most warm” (Arabs: *M* = 3.84, *SD* = 1.49; Asians: *M* = 4.54, *SD* = 1.30; Muslims: *M* = 3.79, *SD* = 1.55).

**Hours of news** Hours of news exposure was assessed by asking participants how many hours they watched or read about the news in the past week (*M* = 5.24, *SD* = 5.13).

**Political conservatism** Political conservatism was assessed using a single-item that asked participants to report their political orientation on a 1 (Liberal) to 7 (Conservative) scale (*M* = 3.62, *SD* = 1.29).

**Religious identification** To assess religious identification, we asked people: “Do you identify with a religion and/or spiritual group?”. For those who identified with a religion, we asked participants to rate on a scale from 1-7 “how important is your religion to how you see yourself?” Those individuals who indicated that they did not belong to a religion were coded as a 0 (*n* = 10,129) on this scale (*M* = 1.79; *SD* = 2.60).

**Age** The mean age of the sample was 47.97 (*SD* = 13.85).

**Education** Education was coded as either no qualification “0” (*n* = 748), Level 1 Certificate “1” (*n* = 2,195), Level 2 Certificate “2” (*n* = 1,037), Level 3 Certificate “3” (*n* = 1,901), Level 4 Certificate “4” (*n* = 862), Level 5 Diploma/Certificate “5” (*n* = 1,279), Level 6 Graduate Certificate/Diploma “6” (*n* = 773), Bachelor’s Degree/Level 7 Diploma/Certificate “7” (*n* = 3,725), Postgraduate Diploma/Certificate “8” (*n* = 1,402), Master’s Degree “9” (*n* = 1,053), or Doctorate Degree “10” (*n* = 296).

**Employment** Employment status was assessed by asking participants if they were currently working. “Yes” was coded as “1” (*n* = 12, 585) and “no” was coded as “0” (*n* = 3,883).

**European ancestry** We also assessed ethic origin, and 15,516 participants indicated that they were of European descent (coded as 1), whereas 1,068 indicated non-European ancestry (coded as 0).

**Gender** The sample included 6,190 males (coded as 1) and 10,391 females (coded as 0).

**Socioeconomic deprivation** The pairwise deleted data set had a mean deprivation index of 4.69 (*SD* = 2.75).

**Parental Status** We assessed parental status by asking participants to indicate their number of children. Participants were coded as “0” if they reported that they do not have children (*n* = 4,331) and “1” if they reported that they do (*n* = 12,253).

**Partner** Participants were asked if they were in a relationship. “Yes” was coded as “1” (*n* = 11,976) and “no” was coded as “0” (*n* = 4,533).

**Urban dwelling** People were coded as either residing in an urban “1” (*n* = 10,856) or rural “0” (*n* = 5,581) area based on New Zealand census data.
