# Supplementary material for: News exposure predicts anti-Muslim prejudice
Source: PLoS One. 2017 Mar 31;12(3):e0174606. doi: 10.1371/journal.pone.0174606 (PMC5375159; doi:10.1371/journal.pone.0174606)
Supplement: S1 Table — (DOCX) [file pone.0174606.s002.docx]

**S1 Table.** Absolute levels of anger toward Asians and Muslims on a scale from 1-7.

| **Anger toward Asians** | | | | | | | | | | | |
| --- | --- | --- | --- | --- | --- | --- | --- | --- | --- | --- | --- |
| **Anger toward Muslims** |  |  |  | | |  |  | | |  |  |
|  |  |  | 1 | 2 | 3 | 4 | 5 | 6 | 7 | NA | Row total |
|  |  | 1 | 4,437 | 177 | 57 | 73 | 25 | 22 | 28 | 8 | 4,827 (34.4%) |
|  |  | 2 | 515 | 1,149 | 161 | 114 | 60 | 24 | 10 | 2 | 2,035 (14.5%) |
|  |  | 3 | 170 | 298 | 429 | 173 | 66 | 24 | 9 | 3 | 1,172 (8.4%) |
|  |  | 4 | 200 | 230 | 221 | 2,484 | 187 | 53 | 17 | 10 | 3,402 (24.3%) |
|  |  | 5 | 159 | 145 | 160 | 371 | 239 | 37 | 10 | 5 | 1,126 (8.0%) |
|  |  | 6 | 86 | 98 | 84 | 182 | 101 | 84 | 17 | 3 | 655 (4.7%) |
|  |  | 7 | 109 | 82 | 61 | 141 | 82 | 54 | 118 | 4 | 651 (4.6%) |
|  |  | NA | 27 | 10 | 10 | 12 | 5 | 1 | 2 | 87 | 154 (1.1%) |
|  |  | Column total | 5,703 (40.7%) | 2,189 (15.6%) | 1,183 (8.4%) | 3,550 (25.3%) | 765 (5.5%) | 299 (2.1%) | 211 (1.5%) | 122 (0.9%) | N=14,022 |
