# Supplementary material for: News exposure predicts anti-Muslim prejudice
Source: PLoS One. 2017 Mar 31;12(3):e0174606. doi: 10.1371/journal.pone.0174606 (PMC5375159; doi:10.1371/journal.pone.0174606)
Supplement: S2 Table — (DOCX) [file pone.0174606.s003.docx]

**S2 Table.** A summary of all variables used in analyses of the pairwise deleted dataset (*N* = 14,022), including means, standard deviations, and ranges.

| **Variable** | **Mean** | **SD** | **Min** | **Max** |
| --- | --- | --- | --- | --- |
| Anger toward Arabs | 2.82 | 1.73 | 1 | 7 |
| Anger toward Asians | 2.51 | 1.57 | 1 | 7 |
| Anger toward Muslims | 2.89 | 1.81 | 1 | 7 |
| Warmth toward Arabs | 3.84 | 1.49 | 1 | 7 |
| Warmth toward Asians | 4.54 | 1.30 | 1 | 7 |
| Warmth toward Muslims | 3.79 | 1.55 | 1 | 7 |
| Hours of news | 5.24 | 5.13 | 0 | 120 |
| Political Conservatism | 3.62 | 1.29 | 1 | 7 |
| Religious Identification | 1.78 | 2.59 | 0 | 7 |
| Age | 47.97 | 13.85 | 18 | 94 |
| Education | 5.92 | 2.84 | 1 | 10 |
| Employed | 0.78 | 0.42 | 0 | 1 |
| European ancestry | 0.94 | 0.23 | 0 | 1 |
| Gender | 0.38 | 0.49 | 0 | 1 |
| Socioeconomic deprivation | 4.69 | 2.75 | 1 | 10 |
| Parent | 0.74 | 0.44 | 0 | 1 |
| Partner | 0.74 | 0.44 | 0 | 1 |
| Urban dwelling | 0.67 | 0.47 | 0 | 1 |
