# Supplementary material for: News exposure predicts anti-Muslim prejudice
Source: PLoS One. 2017 Mar 31;12(3):e0174606. doi: 10.1371/journal.pone.0174606 (PMC5375159; doi:10.1371/journal.pone.0174606)
Supplement: S4 Table — (DOCX) [file pone.0174606.s005.docx]

**S4 Table.** Variance and covariance solutions for geographic regions (n = 67) of a Bayesian regression model of the pairwise deleted dataset (*N* = 14,022) predicting anger toward Arabs, Asians, and Muslims.

|  | **Posterior means** | **95% lower bounds** | **95% upper bounds** |
| --- | --- | --- | --- |
| Var(Arabs)region | 0.001 | 0.000 | 0.003 |
| Var(Asians)region | 0.003 | 0.000 | 0.008 |
| Var(Muslims)region | 0.001 | 0.000 | 0.003 |
| Cov(Arabs,Asians)region | 0.001 | -0.001 | 0.004 |
| Cov(Arabs,Muslims)region | 0.000 | -0.000 | 0.002 |
| Cov(Asians,Muslims)region | 0.000 | -0.002 | 0.003 |
