# Supplementary material for: News exposure predicts anti-Muslim prejudice
Source: PLoS One. 2017 Mar 31;12(3):e0174606. doi: 10.1371/journal.pone.0174606 (PMC5375159; doi:10.1371/journal.pone.0174606)
Supplement: S5 Table — (DOCX) [file pone.0174606.s006.docx]

**S5 Table.** Residual variance structure of a Bayesian regression model of the pairwise deleted dataset (*N* = 14,022) predicting anger toward Arabs, Asians, and Muslims.

|  | **Posterior means** | **95% lower bounds** | **95% upper bounds** |
| --- | --- | --- | --- |
| Var(Arabs)units | 2.850 | 2.783 | 2.918 |
| Var(Asians)units | 2.391 | 2.333 | 2.446 |
| Var(Muslims)units | 3.103 | 3.031 | 3.175 |
| Cov(Arabs,Asians)units | 1.940 | 1.885 | 1.993 |
| Cov(Arabs,Muslims)units | 2.457 | 2.392 | 2.520 |
| Cov(Asians,Muslims)units | 1.776 | 1.720 | 1.829 |
