# Supplementary material for: News exposure predicts anti-Muslim prejudice
Source: PLoS One. 2017 Mar 31;12(3):e0174606. doi: 10.1371/journal.pone.0174606 (PMC5375159; doi:10.1371/journal.pone.0174606)
Supplement: S6 Table — Political conservatism, religious identification, and socioeconomic deprivation were standardized, and age and education were centered. (DOCX) [file pone.0174606.s007.docx]

**S6 Table.** Results of a Bayesian regression model of the pairwise deleted dataset (*N* = 14,022) predicting anger toward Arabs, Asians, and Muslims. Political conservatism, religious identification, and socioeconomic deprivation were standardized, and age and education were centered.

|  | **_Hostility toward Arabs_** | | | | **_Hostility toward Asians_** | | | | **_Hostility toward Muslims_** | | | | |
| --- | --- | --- | --- | --- | --- | --- | --- | --- | --- | --- | --- | --- | --- |
|  | **_Posterior Mean_** | **_95 % Lower Bounds_** | **_95 % Upper Bounds_** | **_pMCMC_** | **_Posterior Mean_** | **_95 % Lower Bounds_** | **_95 % Upper Bounds_** | **_pMCMC_** | **_Posterior Mean_** | **_95 % Lower Bounds_** | **_95 % Upper Bounds_** | **_pMCMC_** |  |
| **_Intercept_** | _3.086_ | _2 .905_ | _3.269_ | _<0.0001***_ | _2.835_ | _2.666_ | _3.003_ | _<0.0001***_ | _3.030_ | _2.845_ | _3.212_ | _<0.0001***_ |  |
| **_Hours of news_** | _0.034_ | _0.004_ | _0.063_ | _0.0264*_ | _0.015_ | _-0.013_ | _0.042_ | _0.2784_ | _0.054_ | _0.023_ | _0.084_ | _0.0008***_ |  |
| **_Political conservatism (standardized)_** | _0.228_ | _0.198_ | _0.258_ | _<0.0001***_ | _0.154_ | _0.126_ | _0.181_ | _<0.0001***_ | _0.249_ | _0.217_ | _0.279_ | _<0.0001***_ |  |
| **_Religious identification (standardized)_** | _-0.103_ | _-0.162_ | _-0.045_ | _0.0002***_ | _-0.083_ | _-0.137_ | _-0.030_ | _0.0026**_ | _-0.085_ | _-0.144_ | _-0.027_ | _0.0032**_ |  |
| **_Age (centered)_** | _0.010_ | _0.007_ | _0.012_ | _<0.0001***_ | _0.002_ | _0.000_ | _0.005_ | _0.0372*_ | _0.010_ | _0.008_ | _0.013_ | _<0.0001***_ |  |
| **_Education (centered)_** | _-0.189_ | _-0.220_ | _-0.160_ | _<0.0001***_ | _-0.163_ | _-0.192_ | _-0.137_ | _<0.0001***_ | _-0.176_ | _-0.208_ | _-0.146_ | _<0.0001***_ |  |
| **_Employed_** | _-0.089_ | _-0.159_ | _-0.018_ | _0.0158*_ | _-0.126_ | _-0.191_ | _-0.061_ | _0.0002**_ | _-0.010_ | _-0.174_ | _-0.025_ | _0.0096**_ |  |
| **_European_** | _-0.076_ | _-0.205_ | _0.047_ | _0.2430_ | _-0.071_ | _-0.185_ | _0.050_ | _0.2386_ | _0.011_ | _-0.125_ | _0.137_ | _0.8818_ |  |
| **_Gender_** | _0.048_ | _-0.010_ | _0.107_ | _0.1106_ | _-0.008_ | _-0.064_ | _0.046_ | _0.7714_ | _0.176_ | _0.117_ | _0.239_ | _<0.0001***_ |  |
| **_Socioeconomic deprivation (standardized)_** | _0.022_ | _-0.007_ | _0.051_ | _0.1372_ | _0.031_ | _0.003_ | _0.058_ | _0.0264*_ | _0.019_ | _-0.011_ | _0.051_ | _0.2260_ |  |
| **_Parent_** | _-0.066_ | _-0.140_ | _0.012_ | _0.0092_^.^ | _-0.048_ | _-0.119_ | _0.020_ | _0.1840_ | _-0.090_ | _-0.166_ | _-0.006_ | _0.0276*_ |  |
| **_Partner_** | _-0.023_ | _-0.092_ | _0.046_ | _0.4996_ | _-0.084_ | _-0.148_ | _-0.022_ | _0.0108*_ | _-0.030_ | _-0.098_ | _0.048_ | _0.4320_ |  |
| **_Urban_** | _-0.013_ | _-0.080_ | _0.053_ | _0.7080_ | _-0.026_ | _-0.100_ | _0.041_ | _0.4586_ | _0.007_ | _-0.062_ | _0.075_ | _0.8302_ |  |
| **_Hours of news X Political conservatism_** | _-0.003_ | _-0.032_ | _0.025_ | _0.7928_ | _0.001_ | _-0.025_ | _0.027_ | _0.9328_ | _-0.016_ | _-0.047_ | _0.012_ | _0.2760_ |  |

Key:

*** pMCMC <.001

** pMCMC <.01

* pMCMC <.05

. pMCMC <.10
