# Supplementary material for: News exposure predicts anti-Muslim prejudice
Source: PLoS One. 2017 Mar 31;12(3):e0174606. doi: 10.1371/journal.pone.0174606 (PMC5375159; doi:10.1371/journal.pone.0174606)
Supplement: S7 Table — (DOCX) [file pone.0174606.s008.docx]

**S7 Table.** Variance and covariance solutions for religious denominations (n = 93) of a Bayesian regression model of the pairwise deleted dataset (*N* = 14,022) predicting warmth toward Arabs, Asians, and Muslims.

|  | **Posterior means** | **95% lower bounds** | **95% upper bounds** |
| --- | --- | --- | --- |
| Var(Arabs)denominations | 0.010 | 0.001 | 0.024 |
| Var(Asians)denominations | 0.004 | 0.000 | 0.012 |
| Var(Muslims)denominations | 0.011 | 0.000 | 0.029 |
| Cov(Arabs,Asians)denominations | 0.004 | -0.001 | 0.012 |
| Cov(Arabs,Muslims)denominations | 0.006 | -0.002 | 0.019 |
| Cov(Asians,Muslims)denominations | 0.003 | -0.002 | 0.012 |
