# Supplementary material for: News exposure predicts anti-Muslim prejudice
Source: PLoS One. 2017 Mar 31;12(3):e0174606. doi: 10.1371/journal.pone.0174606 (PMC5375159; doi:10.1371/journal.pone.0174606)
Supplement: S9 Table — (DOCX) [file pone.0174606.s010.docx]

**S9 Table.** Residual variance structure of a Bayesian regression model of the pairwise deleted dataset (*N* = 14,022) predicting warmth toward Arabs, Asians, and Muslims.

|  | **Posterior means** | **95% lower bounds** | **95% upper bounds** |
| --- | --- | --- | --- |
| Var(Arabs)units | 2.069 | 2.020 | 2.118 |
| Var(Asians)units | 1.643 | 1.604 | 1.681 |
| Var(Muslims)units | 2.202 | 2.151 | 2.255 |
| Cov(Arabs,Asians)units | 1.156 | 1.119 | 1.191 |
| Cov(Arabs,Muslims)units | 1.753 | 1.707 | 1.798 |
| Cov(Asians,Muslims)units | 1.106 | 1.070 | 1.143 |
