# Supplementary material for: News exposure predicts anti-Muslim prejudice
Source: PLoS One. 2017 Mar 31;12(3):e0174606. doi: 10.1371/journal.pone.0174606 (PMC5375159; doi:10.1371/journal.pone.0174606)
Supplement: S10 Table — Political conservatism, religious identification, and socioeconomic deprivation were standardized, and age and education were centered. (DOCX) [file pone.0174606.s011.docx]

**S10 Table.** Results of a Bayesian regression model of the pairwise deleted dataset (*N* = 14,022) predicting wamth toward Arabs, Asians, and Muslims. Political conservatism, religious identification, and socioeconomic deprivation were standardized, and age and education were centered.

|  | **_Acceptance of Arabs_** | | | | **_Acceptance of Asians_** | | | | | **_Acceptance of Muslims_** | | | | |
| --- | --- | --- | --- | --- | --- | --- | --- | --- | --- | --- | --- | --- | --- | --- |
|  | **_Posterior Mean_** | **_95 % Lower Bounds_** | **_95 % Upper Bounds_** | **_pMCMC_** | **_Posterior Mean_** | **_95 % Lower Bounds_** | **_95 % Upper Bounds_** | **_pMCMC_** | **_Posterior Mean_** | | **_95 % Lower Bounds_** | **_95 % Upper Bounds_** | **_pMCMC_** |  |
| **_Intercept_** | _3 .712_ | _3.545_ | _3.868_ | _<0.0001***_ | _4.460_ | _4.324_ | _4.602_ | _<0.0001***_ | _3.727_ | | _3.558_ | _3.885_ | _<0.0001***_ |  |
| **_Hours of news_** | _-0.021_ | _-.0479_ | _0.002_ | _0.0918_^.^ | _0.005_ | _-0.017_ | _0.027_ | _0.6930_ | _-0.039_ | | _-0.054_ | _-0.013_ | _0.0028**_ |  |
| **_Political conservatism (standardized)_** | _-0.235_ | _-0.261_ | _-0.210_ | _<0.0001***_ | _-0.125_ | _-0.147_ | _-0.102_ | _<0.0001***_ | _-0.269_ | | _-0.300_ | _-0.242_ | _<0.0001***_ |  |
| **_Religious identification (standardized)_** | _0.148_ | _0.091_ | _0.199_ | _<0.0001***_ | _0.126_ | _0.082_ | _0.173_ | _<0.0001***_ | _0.093_ | | _0.039_ | _0.149_ | _0.0004***_ |  |
| **_Age (centered)_** | _-0.007_ | _-0.009_ | _-0.005_ | _<0.0001***_ | _0.004_ | _0.002_ | _0.006_ | _<0.0001***_ | _-0.009_ | | _-0.011_ | _-0.007_ | _<0.0001***_ |  |
| **_Education (centered)_** | _0.158_ | _0.134_ | _0.185_ | _<0.0001***_ | _0.115_ | _0.094_ | _0.139_ | _<0.0001***_ | _0.181_ | | _0.155_ | _0.2078_ | _<0.0001***_ |  |
| **_Employed_** | _0.044_ | _-0.017_ | _0.104_ | _0.1582_ | _0.001_ | _-0.053_ | _0.054_ | _0.9710_ | _0.098_ | | _0.038_ | _0.162_ | _0.0012**_ |  |
| **_European_** | _-0.034_ | _-0.140_ | _0.073_ | _0.5446_ | _-0.016_ | _-0.114_ | _0.078_ | _0.7606_ | _-0.032_ | | _-0.142_ | _0.080_ | _0.5796_ |  |
| **_Gender_** | _-0.078_ | _-0.128_ | _-0.027_ | _0.0018**_ | _-0.058_ | _-0.103_ | _-0.014_ | _0.009**_ | _-0.202_ | | _-0.254_ | _-0.152_ | _<0.0001***_ |  |
| **_Socioeconomic deprivation (standardized)_** | _0.010_ | _-0.017_ | _0.035_ | _0.4470_ | _-0.015_ | _-0.038_ | _0.008_ | _0.2324_ | _0.004_ | | _-0.023_ | _0.031_ | _0.7254_ |  |
| **_Parent_** | _0.008_ | _-0.056_ | _0.074_ | _0.8156_ | _-0.023_ | _-0.079_ | _0.037_ | _0.4482_ | _0.045_ | | _-0.019_ | _0.115_ | _0.1948_ |  |
| **_Partner_** | _0.020_ | _-0.041_ | _0.078_ | _0.5210_ | _0.051_ | _-0.003_ | _0.104_ | _0.0636_^.^ | _-0.002_ | | _-0.063_ | _0.058_ | _0.9546_ |  |
| **_Urban_** | _0.099_ | _0.009_ | _0.185_ | _0.0266*_ | _0.072_ | _-0.013_ | _0.155_ | _0.0592_^.^ | _0.072_ | | _-0.013_ | _0.155_ | _0.0958_^.^ |  |
| **_Hours of news X Political conservatism_** | _0.003_ | _-0.022_ | _0.026_ | _0.8388_ | _0.009_ | _-0.013_ | _0.030_ | _0.4406_ | _0.002_ | | _-0.022_ | _0.027_ | _0.8688_ |  |

Key:

*** pMCMC <.001

** pMCMC <.01

* pMCMC <.05

. pMCMC <.10
