# Supplementary material for: News exposure predicts anti-Muslim prejudice
Source: PLoS One. 2017 Mar 31;12(3):e0174606. doi: 10.1371/journal.pone.0174606 (PMC5375159; doi:10.1371/journal.pone.0174606)
Supplement: S11 Table — (DOCX) [file pone.0174606.s012.docx]

**S11 Table.** Variance and covariance solutions for religious denominations (n = 93) of a Bayesian regression model of the Ameila imputed dataset (*N* = 16,548) predicting anger toward Arabs, Asians, and Muslims.

|  | **Posterior means** | **95% lower bounds** | **95% upper bounds** |
| --- | --- | --- | --- |
| **Var(Arabs)denominations** | 0.023 | 0.007 | 0.065 |
| **Var(Asians)denominations** | 0.012 | 0.003 | 0.039 |
| **Var(Muslims)denominations** | 0.011 | 0.002 | 0.040 |
| **Cov(Arabs,Asians)denominations** | 0.010 | 0.000 | 0.037 |
| **Cov(Arabs,Muslims)denominations** | 0.012 | 0.002 | 0.044 |
| **Cov(Asians,Muslims)denominations** | 0.007 | -0.001 | 0.027 |
