# Supplementary material for: News exposure predicts anti-Muslim prejudice
Source: PLoS One. 2017 Mar 31;12(3):e0174606. doi: 10.1371/journal.pone.0174606 (PMC5375159; doi:10.1371/journal.pone.0174606)
Supplement: S12 Table — (DOCX) [file pone.0174606.s013.docx]

**S12 Table.** Variance and covariance solutions for geographic regions (n = 67) of a Bayesian regression model of the Ameila imputed dataset (*N* = 16,548) predicting anger toward Arabs, Asians, and Muslims.

|  | **Posterior means** | **95% lower bounds** | **95% upper bounds** |
| --- | --- | --- | --- |
| **Var(Arabs)region** | 0.000 | 0.000 | 0.005 |
| **Var(Asians)region** | 0.002 | 0.000 | 0.007 |
| **Var(Muslims)region** | 0.000 | 0.000 | 0.005 |
| **Cov(Arabs,Asians)region** | 0.000 | 0.000 | 0.004 |
| **Cov(Arabs,Muslims)region** | 0.000 | 0.000 | 0.003 |
| **Cov(Asians,Muslims)region** | 0.000 | -0.001 | 0.003 |
