# Supplementary material for: News exposure predicts anti-Muslim prejudice
Source: PLoS One. 2017 Mar 31;12(3):e0174606. doi: 10.1371/journal.pone.0174606 (PMC5375159; doi:10.1371/journal.pone.0174606)
Supplement: S13 Table — (DOCX) [file pone.0174606.s014.docx]

**S13 Table.** Residual variance structure of a Bayesian regression model of the Ameila imputed dataset (*N* = 16,548) predicting anger toward Arabs, Asians, and Muslims.

|  | **Posterior means** | **95% lower bounds** | **95% upper bounds** |
| --- | --- | --- | --- |
| **Var(Arabs)units** | 2.914 | 2.851 | 2.977 |
| **Var(Asians)units** | 2.450 | 2.398 | 2.504 |
| **Var(Muslims)units** | 3.178 | 3.110 | 3.248 |
| **Cov(Arabs,Asians)units** | 1.971 | 1.921 | 2.022 |
| **Cov(Arabs,Muslims)units** | 2.500 | 2.441 | 2.561 |
| **Cov(Asians,Muslims)units** | 2.450 | 2.398 | 2.504 |
