# Supplementary material for: News exposure predicts anti-Muslim prejudice
Source: PLoS One. 2017 Mar 31;12(3):e0174606. doi: 10.1371/journal.pone.0174606 (PMC5375159; doi:10.1371/journal.pone.0174606)
Supplement: S14 Table — (DOCX) [file pone.0174606.s015.docx]

**S14 Table.** Variance and covariance solutions for religious denominations (n = 93) of a Bayesian regression model of the Ameila imputed dataset (*N* = 16,548) predicting warmth toward Arabs, Asians, and Muslims.

|  | **Posterior means** | **95% lower bounds** | **95% upper bounds** |
| --- | --- | --- | --- |
| **Var(Arabs)denominations** | 0.010 | 0.003 | 0.033 |
| **Var(Asians)denominations** | 0.006 | 0.001 | 0.022 |
| **Var(Muslims)denominations** | 0.009 | 0.002 | 0.031 |
| **Cov(Arabs,Asians)denominations** | 0.004 | 0.000 | 0.018 |
| **Cov(Arabs,Muslims)denominations** | 0.006 | 0.000 | 0.022 |
| **Cov(Asians,Muslims)denominations** | 0.004 | -0.001 | 0.015 |
