# Supplementary material for: News exposure predicts anti-Muslim prejudice
Source: PLoS One. 2017 Mar 31;12(3):e0174606. doi: 10.1371/journal.pone.0174606 (PMC5375159; doi:10.1371/journal.pone.0174606)
Supplement: S15 Table — (DOCX) [file pone.0174606.s016.docx]

**S15 Table.** Variance and covariance solutions for geographic regions (n = 67) of a Bayesian regression model of the Ameila imputed dataset (*N* = 16,548) predicting warmth toward Arabs, Asians, and Muslims.

|  | **Posterior means** | **95% lower bounds** | **95% upper bounds** |
| --- | --- | --- | --- |
| Var(Arabs)region | 0.009 | 0.003 | 0.019 |
| Var(Asians)region | 0.006 | 0.002 | 0.015 |
| Var(Muslims)region | 0.009 | 0.003 | 0.019 |
| Cov(Arabs,Asians)region | 0.009 | 0.003 | 0.019 |
| Cov(Arabs,Muslims)region | 0.008 | 0.003 | 0.017 |
| Cov(Asians,Muslims)region | 0.005 | 0.001 | 0.012 |
