# Supplementary material for: News exposure predicts anti-Muslim prejudice
Source: PLoS One. 2017 Mar 31;12(3):e0174606. doi: 10.1371/journal.pone.0174606 (PMC5375159; doi:10.1371/journal.pone.0174606)
Supplement: S16 Table — (DOCX) [file pone.0174606.s017.docx]

**S16 Table.** Residual variance structure of a Bayesian regression model of the Ameila imputed dataset (*N* = 16,548) predicting warmth toward Arabs, Asians, and Muslims.

|  | **Posterior means** | **95% lower bounds** | **95% upper bounds** |
| --- | --- | --- | --- |
| Var(Arabs)units | 2.117 | 2.071 | 2.163 |
| Var(Asians)units | 1.691 | 1.655 | 1.728 |
| Var(Muslims)units | 2.249 | 2.201 | 2.299 |
| Cov(Arabs,Asians)units | 1.186 | 1.152 | 1.220 |
| Cov(Arabs,Muslims)units | 1.783 | 1.741 | 1.827 |
| Cov(Asians,Muslims)units | 1.132 | 1.098 | 1.167 |
